# Determinants of Sexual Activity and Pregnancy among Unmarried Young Women in Urban Kenya: A Cross-Sectional Study

**DOI:** 10.1371/journal.pone.0129286

**Published:** 2015-06-05

**Authors:** Chinelo C. Okigbo, Ilene S. Speizer

**Affiliations:** 1 Department of Maternal and Child Health, Gillings School of Global Public Health, University of North Carolina, Chapel Hill, North Carolina, United States of America; 2 Measurement, Learning, and Evaluation Project, Carolina Population Center, University of North Carolina, Chapel Hill, North Carolina, United States of America; University of Vienna, AUSTRIA

## Abstract

**Objectives:**

With age of marriage rising in Kenya, the period between onset of puberty and first marriage has increased, resulting in higher rates of premarital sexual activity and pregnancy. We assessed the determinants of sexual activity and pregnancy among young unmarried women in urban Kenya.

**Methods:**

Baseline data from five urban areas in Kenya (Nairobi, Mombasa, Kisumu, Machakos, and Kakamega) collected in 2010 by the Measurement, Learning & Evaluation project were used. Women aged 15-24 years, who had never been married, and were not living with a male partner at the time of survey (weighted n=2020) were included. Using weighted, multivariate Cox proportional hazard regression and logistic regression analyses, we assessed factors associated with three outcome measures: time to first sex, time to first pregnancy, and teenage pregnancy.

**Results:**

One-half of our sample had ever had sex; the mean age at first sex among the sexually-experienced was 17.7 (± 2.6) years. About 15% had ever been pregnant; mean age at first pregnancy was 18.3 (±2.2) years. Approximately 11% had a teenage pregnancy. Three-quarters (76%) of those who had ever been pregnant (weighted n=306) reported the pregnancy was unwanted at the time. Having secondary education was associated with a later time to first sex and first pregnancy. In addition, religion, religiosity, and employment status were associated with time to first sex while city of residence, household size, characteristics of household head, family planning knowledge and misconceptions, and early sexual debut were significantly associated with time to first pregnancy. Education, city of residence, household wealth, early sexual debut, and contraceptive use at sexual debut were associated with teenage pregnancy for those 20-24 years.

**Conclusion:**

Understanding risk and protective factors of youth sexual and reproductive health can inform programs to improve young people’s long-term potential by avoiding early and unintended pregnancies.

## Introduction

The mortality rate among young people (ages 15–24) is highest in Africa compared to other regions of the world; and within Africa, young women are more likely to die compared to their male counterparts. [1] Pregnancy-related complications are the major cause of mortality and morbidity among young African women and have also been associated with poor child health outcomes. [1–3] The intention and timing of a pregnancy play major roles on its outcome. It has been found that, compared to intended pregnancies, unintended pregnancies have higher rates of adverse health outcomes including complications from unsafe abortion. [4, 5] It is, therefore, logical that a reduction in unintended pregnancy rates will contribute to decreased morbidity and mortality for both the mother and the child. [4–7] Likewise, early (adolescent) pregnancy, regardless of its intention, has been associated with higher risks of adverse maternal and child health outcomes such as pre-eclampsia and preterm delivery. [8] Though many pregnancies among young women occur within marital unions, the rising age at first marriage in many sub-Saharan African countries has led to an increased period of time between onset of puberty and first marriage, further increasing the likelihood of premarital sex and unintended pregnancies. [3, 9, 10] Premarital sexual behaviors such as early sexual debut and pregnancy are stigmatized in many sub-Saharan African settings as they are viewed to deviate from the norm, in that they are expected to occur within marital unions. [11] Unmarried young women who are sexually active are often in relationships where they lack power due to having older and/or wealthier male partners. [11–13] Being in such relationships decreases their probability of having protected sex, thereby increasing their risk of unintended pregnancy and sexually transmitted infections (STIs) including HIV. [12] Therefore, unmarried young women are at a greater risk as compared to married and/or older women for unintended pregnancy, unsafe abortion, and STIs. [7, 13–18] Premarital sexual activity and consequently pregnancy have socioeconomic consequences such as decreased probability of completing education, decreased probability of gainful participation in the workforce, lower social standing, and increased dependency on social welfare programs. [3, 7, 10] Therefore, understanding the risk and protective factors of premarital sexual and reproductive behaviors is relevant to programs and policies targeted at improving youth development and health.

Evidence emanating from Kenya suggests that many young women are sexually-experienced, with 16 years being the average age at first sex. [18–22] According to a 2009 study, among never-married young women (ages 15–24) living in Kenya, about half (47%) had ever had sex. [23] In addition, studies have shown that the majority (>70%) of sexually-active youth do not practice protected sex [10, 24, 25] and as a result are at a risk of premarital pregnancy. About 20–50% of adolescents in Kenya had ever been pregnant [10, 20, 24] with a median age at first pregnancy of 18.5 years. [21] In a 2013 study in Nairobi, Kenya, about a quarter (24%) of women aged 15–49 years had ever experienced an unintended pregnancy; the authors found that adolescents together with unmarried women were more likely to have had an unintended pregnancy compared to older and married women. [7] Ngom and colleagues in 2003 found that 11% of never-married 15–19 year old girls in Nairobi had ever experienced an unwanted pregnancy. [25] Unfortunately, many of these unmarried young women face high unmet need for contraception further predisposing them to unintended pregnancy and risk of unsafe abortion. [3, 24] According to Khan and Mishra (2008), 40% of sexually-active unmarried young Kenyan women had an unmet need for family planning in 2003. [24]

In this study, we aimed to examine the individual and household factors associated with timing of first sex and first pregnancy among unmarried young women in urban Kenya. The rationale is that if we understand the factors associated with these behaviors, we can better plan and implement policies and programs targeted at improving the sexual and reproductive health of young women in urban Kenya. This study is informed by the Problem-Behavior Theory developed by Jessor in the 1960s, which has been widely applied to research on adolescent health behaviors such as alcohol/drug use and risky sexual behaviors. [26, 27] The theory posits that it is the balance between the risk and protective factors that exist within a young person’s environment and the young person’s personality that determines his/her likelihood of engaging in a problem behavior. A problem behavior, according to Jessor, is defined socially according to norms of the environment or the institutions of authority in which the young person is situated. [26, 27] In this study, the risk factors are hypothesized to provide an enabling environment for the young woman to engage in risky sexual behaviors while the protective factors help the young woman to deter from such behaviors. The risky sexual behaviors and outcomes included in this study are premarital sexual activity, premarital pregnancy, and teenage pregnancy. The Problem-Behavior Theory has been applied in our study setting and found to provide explanatory account of youth problem behaviors. [28] Studies that applied this theory have demonstrated that demographic and socioeconomic factors are associated with youth’s engagement in risky sexual behaviors. [11, 18, 20, 25, 28–31] These factors include age, education, marital status, religion, urban vs. rural residence, wealth, employment, and migration status. These studies were either national or community-based surveys involving both male and female youth in rural and urban areas regardless of their marital status. Our study focused on unmarried women aged 15–24 living in five urban areas in Kenya. We examined how individual and household factors are associated with transition to first sex and first pregnancy. We also assessed the association between these factors and teenage pregnancy among a sub-sample of young women aged 20–24 years. Our results will contribute to the growing evidence of factors that predispose young women to poor reproductive health outcomes.

## Methods

### Data and Sample

This study uses Kenya baseline survey data collected by the Measurement, Learning & Evaluation (MLE) project in 2010. [32] The MLE project is the evaluation arm of a four-country Urban Reproductive Health Initiative (URHI), a Bill & Melinda Gates Foundation funded project. [33] The Kenya URHI is also known as Tupange (Swahili for “Let’s Plan”) and aims to increase contraceptive prevalence in five urban sites—Nairobi, Mombasa, Kisumu, Machakos, and Kakamega. The Kenya URHI chose these urban areas based on a prior needs assessment. Though not all highly populated urban areas were selected, we believe that the selected sites provide a good representation of urban areas in Kenya as they include the two largest urban areas (Nairobi and Mombasa) as well as three other highly populated cities (Kisumu, Machakos, and Kakamega). A two-stage cluster sampling design was used in each site to select a representative sample of reproductive-age women (15–49 years) at the household level. The 2009 Kenya Population and Housing Census was used as the sampling frame. In the first stage, a random sample of 74 primary sampling units (PSU) was selected in each site except for Nairobi which was oversampled with 142 PSUs. In the second stage, a random sample of 30 households per PSU was selected for inclusion in the study. All women aged 15–49 years in each selected household were asked to participate in the study. Upon receipt of informed consent, eligible women were interviewed by a female interviewer in one of the four commonly spoken local languages (Kiswahili, Luhya, Kamba, or Dholuo) using a paper-and-pencil questionnaire. Interviews were conducted in a location within or close to the woman’s household with some level of privacy. Interviews were conducted at times convenient to the women, mostly in the evenings and on the weekends. Out of 10502 eligible women, 8932 of them were surveyed, a response rate of 85%. More information on the study design, recruitment strategies, and key findings of the baseline survey can be found in the baseline report. [32] The data is available upon request; request should be made through the project’s website. [33]

For this analysis, the sample was restricted to unmarried young women. A total of 3619 women aged 15–24 years were interviewed at baseline (41% of the full sample). Of these, some were dropped from the analysis because they were married or living with a male partner (n = 1659) or had missing data on any of the key exposure variables (n = 10). There were no missing data on the outcome variables. These restrictions yielded an analytical sample of 1948 women aged 15–24 years (weighted n = 2020). The city-level sample sizes are shown in Table 1. According to the 2009 Kenya Population and Housing Census [34], 15–24 year old women make up approximately 42% of reproductive-age women living in urban areas of Kenya, which makes our sample representative of young women in urban Kenya.

**Table 1:** Descriptive characteristics of never-married women aged 15–24 years in urban Kenya.

Characteristics	Total; N (%)	Age Groups	
		15–19 years; N (%)	20–24 years; N (%)
**Sociodemographic factors**			
**Education** *			
Primary or less	705 (34.9)	385 (47.3)	319 (26.5)
Secondary	898 (44.5)	376 (46.0)	523 (43.4)
Higher	417 (20.6)	54 (6.7)	363 (30.1)
**Religion** *			
Other Christians	1282 (63.5)	446 (54.7)	836 (69.4)
Catholic	410 (20.3)	164 (20.1)	246 (20.4)
Muslim/Others	328 (16.2)	205 (25.2)	123 (10.2)
**Religiosity**			
Somewhat/Not religious	896 (44.4)	383 (47.0)	513 (42.6)
Strongly religious	1124 (55.6)	432 (53.0)	692 (57.4)
**Employment Status** *			
Not employed	467 (23.1)	234 (28.7)	233 (19.3)
Student	658 (32.6)	409 (50.2)	249 (20.7)
Employed	895 (44.3)	172 (21.2)	722 (60.0)
**Household factors**			
**City where household is located** *			
Nairobi	1400 (69.3)	487 (59.7)	913 (75.8)
Mombasa	455 (22.6)	241 (29.6)	214 (17.8)
Kisumu	99 (4.9)	55 (6.7)	44 (3.7)
Machakos	35 (1.7)	18 (2.2)	17 (1.3)
Kakamega	31 (1.5)	14 (1.8)	17 (1.4)
**Number of people living in household** *			
1–5 persons	1302 (64.5)	429 (52.6)	873 (72.5)
6 or more persons	718 (35.5)	386 (47.4)	332 (27.5)
**Household wealth quintile**			
Poorest	296 (14.7)	119 (14.6)	177 (14.7)
Poor	382 (18.9)	126 (15.4)	256 (21.3)
Middle	261 (12.9)	127 (15.5)	134 (11.1)
Rich	416 (20.6)	197 (24.1)	219 (18.2)
Richest	665 (32.9)	248 (30.4)	417 (34.7)
**Head of household** *			
Father figure	1037 (51.3)	520 (63.8)	516 (42.8)
Mother figure	638 (31.6)	265 (32.5)	373 (31.0)
Self	345 (17.1)	30 (3.7)	315 (26.2)
**Duration of residence in household** *			
One year or less	1065 (52.7)	364 (44.7)	701 (58.2)
2–5 years	491 (24.3)	192 (23.5)	299 (24.8)
Six or more years	464 (23.0)	259 (31.8)	205 (17.0)
**Family planning knowledge factors**			
**Knowledge of contraceptive methods** *			
Yes	1699 (84.1)	586 (71.9)	1113 (92.3)
No	321 (15.9)	229 (28.1)	92 (7.7)
**Family Planning misconceptions** *			
Low level (score: 0–2)	622 (30.8)	348 (42.7)	274 (22.7)
Medium level (score: 3–5)	544 (26.9)	219 (25.7)	335 (27.8)
High level (score: 6–8)	854 (42.3)	258 (31.6)	596 (49.5)
**Weighted N**	**2020**	**815**	**1205**

* Distributional differences between the age groups are statistically significant at p-value <0.05

Ethical approval for this study was obtained from the University of North Carolina at Chapel Hill Institutional Review Board and the Kenya Medical Research Institute. Verbal informed consent was obtained from all respondents prior to study participation. Verbal, instead of written, informed consent was obtained from the study participants because of the sensitive nature of the questions asked, such as information on sexual and reproductive behaviors. Young women aged less than 18 years were considered emancipated adults for the same reason and were able to provide their own consent. The interviewers documented the receipt of verbal informed consent on the individual consent form. This method of obtaining informed consent from all respondents including those aged less than 18 years was approved for the overall study and for this analysis by the two ethical committee boards.

### Measures

The outcome variables are as follows: 1) time to first sex; 2) time to first pregnancy; and 3) experience of a teenage pregnancy. The first outcome variable, time to first sex, was assessed using the question “*How old were you when you had sexual intercourse for the very first time*?” Those who had not had sexual activity at the time of survey were right-censored. The second outcome variable, time to first pregnancy, was assessed using the question “*How old were you when you got pregnant the very first time*?” This question was asked irrespective of the outcome of the first pregnancy (livebirth, abortion, miscarriage, or stillbirth). Those who had never been pregnant at the time of survey were also right-censored. The third outcome variable, experience of a teenage pregnancy, was assessed only among those aged 20–24 years (weighted n = 1205). This restriction is to eliminate a potential bias of including respondents who are teenagers and have the potential to still experience a teenage pregnancy. The 20–24 year olds subsample is homogenous i.e. they have all exited teenage years, hence they have exhausted their potential to experience a teenage pregnancy. The ‘experience of a teenage pregnancy’ variable was created as a binary variable: women aged 20–24 who had their first pregnancy at or before age 19 were coded ‘1’ and those who had their first pregnancy at or after age 20 together with those who had never been pregnant at the time of survey were coded ‘0’.

The exposure variables are grouped into sociodemographic factors, household factors, family planning knowledge-related factors, and sexual/contraceptive behavior factors (see Tables 1–3 for variables and their coding). The sociodemographic factors include education (primary or less, secondary or higher education), religion (Catholic, other Christians, Muslim and others), religiosity (strongly religious, somewhat or not religious), and employment status (employed, student or not employed). The household factors include city of residence (Nairobi, Mombasa, Kisumu, Machakos, and Kakamega), number of people living in the household (1–3, 4–6, 7 or more persons), head of household (self, father, or mother), and duration of residence in the household (one year or less, 2–5 years, 6 or more years). Also included in the household characteristics is the household wealth index (poorest, poor, middle, rich, richest); this was calculated using the principal component analysis of ownership of household items such as television, phone, motor vehicle, drinking water source, and toilet facilities to mention a few. This method of measuring household wealth has been validated in several settings including Kenya. [35] Two family planning knowledge factors were assessed. The first one is the knowledge of family planning methods. This variable was assessed using the question “*Which methods have you heard of*”. The variable was coded ‘1’ if the respondent spontaneously mentioned any of the following methods: female sterilization, male sterilization, daily pills, intrauterine device, injections, implants, male condoms, female condoms, rhythm method, withdrawal, emergency contraceptive pills, lactational amenorrhea, spermicides or diaphragm. The variable was coded ‘0’ otherwise. The second family planning knowledge factor was assessed using questions that asked the respondents about their belief in misconceptions about family planning. The family planning misconception variable was created using an eight-item scale that included statements on the popular misconceptions about family planning (see S1 Table). The score for this scale ranged from 0–8 and was grouped into three categories: low level (score of 0–2), medium level (score of 3–5) and high level (score of 6–8) of belief in family planning misconceptions. The sexual and contraceptive behavior variables include: i) early sexual debut defined as age at first sex on or before age 14; and ii) use of a modern contraceptive method at sexual debut. The modern contraceptive methods assessed include the following: female sterilization, male sterilization, daily pills, intrauterine device, injections, implants, male condoms, female condoms, emergency contraceptive pills, lactational amenorrhea, spermicides or diaphragm.

**Table 2:** Prevalence of sexual and reproductive behaviors among never-married women aged 15–24 years in urban Kenya.

	Total; N (%)	Age Groups	
		15–19 years; N (%)	20–24 years; N (%)
**Ever had sex** *			
Yes	1019 (50.4)	277 (34.0)	742 (61.5)
No	1001 (49.6)	538 (66.0)	463 (38.5)
**Early sexual debut **(age at first sex≤14 years) ^Ɨ^ *			
Yes	92 (9.0)	58 (20.9)	34 (4.5)
No	927 (91.0)	220 (79.1)	707 (95.5)
Mean age at first sex (SD)*	17.7 (2.6)	15.8 (2.5)	18.5 (2.2)
**Ever been pregnant** *			
Yes	306 (15.2)	65 (8.0)	241 (20.0)
No	1714 (84.8)	750 (92.0)	964 (80.0)
Mean age at first pregnancy (SD)^β^ *	18.3 (2.2)	16.4 (1.4)	18.8 (2.0)
**Period between first sex and first pregnancy** ^β^ *			
Mean (SD) in years	1.3 (1.8)	0.6 (0.8)	1.5 (1.8)
**Intention of first pregnancy** ^β^			
Wanted at the time	73 (23.9)	13 (19.7)	60 (25.1)
Not wanted at the time	234 (76.1)	53 (80.3)	181 (74.9)
**Teenage pregnancy**			
Yes	213 (10.6)	65 (8.1)	148 (12.3)
No	1807 (89.4)	750 (91.9)	1057 (87.7)
**Intention of teenage pregnancy** ^ψ^			
Wanted at the time	44 (20.8)	13 (19.7)	31 (21.3)
Not wanted at the time	169 (79.2)	53 (80.3)	116 (78.7)
**Weighted N**	**2020**	**815**	**1205**

* Distributional differences between the age groups are statistically significant at p-value <0.05

SD: Standard deviation

^Ɨ^ Among those who had ever had sex, weighted n = 1019

^β^ Among those who had ever been pregnant, weighted n = 307

^ψ^ Among those who had ever had teenage pregnancy, weighted n = 213

**Table 3:** Prevalence of contraceptive behaviors among never-married, sexually-experienced females aged 15–24 years in urban Kenya.

	Total; N (%)	Age Groups	
		15–19 years; N (%)	20–24 years; N (%)
Modern contraceptive use at sexual debut	307 (30.1)	68 (24.4)	239 (32.2)
Male condom use at sexual debut^α^	238 (77.7)	58 (85.6)	180 (75.4)
Current modern FP method use*	352 (34.6)	70 (25.2)	282 (38.1)
Current male condom use ^δ^	223 (63.3)	48 (69.0)	175 (61.9)
Current modern LARC use ^δ^	20 (5.8)	3 (5.0)	17 (6.0)
**Weighted N**	**1019**	**277**	**742**

^α^ Among those who used a contraceptive method at sexual debut, weighted n = 307

^δ^ Among those currently using a modern method, weighted n = 352

* Distributional differences between the age groups are statistically significant at p-value <0.05

### Statistical analysis and analytical consideration

All analyses were weighted based on probability of selection, response rates, and adjusted for the clustered survey design. This was done using the ‘*svy*’ function in Stata version 13. [36] Descriptive statistics (proportions and means) are presented in Tables 1–3 to demonstrate differences between age groups, i.e. 15–19 years versus 20–24 years, where appropriate. The statistical significance of the age group differences was assessed using the Rao-Scott correction F statistic for Pearson chi-square and Wald tests provided by the ‘*svy*’ function in Stata. We acknowledged in the results discussion that some of the statistical significance observed between age groups is due to the fact that the younger age group is yet to exhaust their opportunity of experiencing the outcomes. Cox proportional hazards regression models of ‘time to first sex’ and ‘time to first pregnancy’ were conducted and the goodness-of-fit tests of the models assessed. We ran multiple models including the exposure variables one at a time to ensure that they added to the fit of the models; the Cox models presented are the full adjusted models. Multivariate logistic regression of ‘experience of teenage pregnancy’ among the 20–24 year olds was also conducted together with the goodness-of-fit test of the model.

## Results

Table 1 shows the descriptive characteristics of our full sample and by age group (i.e. 15–19 year olds versus 20–24 year olds). About 60% of our unmarried youth sample was aged 20–24 years. A majority of our sample had at least secondary education with 7% of the 15–19 year old reporting higher education compared to 30% of the 20–24 year olds. One-fifths of our sample is of the Catholic denomination, 64% are of other Christian denomination, while the rest are Muslims or another/no religion. Slightly more than half of our sample (56%) reported being strongly religious. As expected, a higher proportion of respondents aged 15–19 years were students (50%) and unemployed (29%) compared to those aged 20–24 years who were mainly employed (60%). A majority (69%) of our sample resides in Nairobi, the capital city of Kenya; this is expected as Nairobi was oversampled given that it is the largest city in Kenya. One-third (36%) of our sample live in households with six or more people; those aged 20–24 years were more likely to come from a smaller household compared to those aged 15–19 years (73% vs. 53%). Many of the respondents live in a parent-headed household; however, those aged 20–24 were more like to report being the head of the household compared to those aged 15–19 (26% vs. 4%). About one-quarter of our sample had lived in the same household for more than six years, though those aged 20–24 years were less likely to have done so compared to those aged 15–19 years (17% vs. 32%). Also included in Table 1 are the proportions of young women with family planning knowledge factors. The overwhelming majority (84%) of our sample spontaneously mentioned at least one of the many contraceptive methods. However, there is a high prevalence of misconceptions about family planning in our sample. About 42% of our sample had a high score (6–8) indicating a high level of misconceptions; this value differed by age group as 32% of 15–19 year olds compared to 50% of 20–24 year olds had high levels of belief in family planning misconceptions. In contrast, 43% of the 15–19 year olds had a low score (0–3) compared to 23% of 20–24 year olds.

The prevalence of sexual and reproductive health behaviors among our unmarried youth sample is presented in Table 2. About half (50%) of our sample had ever had sex; one in three of 15–19 year olds compared to two in three of the 20–24 year olds reported ever having sex. The mean age at first sex is 17.7 years (median = 17 years); as expected, the mean age is lower for those aged 15–19 (mean = 15.8 years, median = 16 years) and slightly higher for those aged 20–24 years (mean = 18.5 years, median = 19 years). About 9% of the sample reported early sexual debut, that is, age at first sex was 14 years or younger. About 15% of the sample had ever been pregnant; as expected, a higher proportion (20%) of those aged 20–24 were ever pregnant than those aged 15–19 (8%). The mean age at first pregnancy is 18.3 years with a standard deviation of 2.2 years (median = 18 years). The mean age at first pregnancy was lower among the younger youth who had ever been pregnant at 16.4 years (median = 16 years) compared to the older youth at 18.8 years (median = 19 years). The time period between age at first sex and age at first pregnancy among our sample who had ever been pregnant (weighted n = 307) ranged from 0–10 years with a mean of 1.3 years (median = 1 year) and a standard deviation of 1.8 years. Among those who reported ever being pregnant, about three-quarters reported that their first pregnancy was unwanted at the time. There was no difference in the prevalence of unwanted pregnancy by age group. We also assessed the prevalence of teenage pregnancy in our sample and found that 11% of our sample had experienced a teenage pregnancy with about 12% of the 20–24 year olds and only 8% of the 15–19 year olds reporting a teenage pregnancy. A majority (79%) of the reported teenage pregnancies was unwanted at the time; there was no significant difference by age.

We assessed the prevalence of contraceptive behaviors among our sexually-experienced unmarried youth sample (weighted n = 1019; see Table 3) and found that only about a third (30%) of those who reported ever having sex used a modern contraceptive method at first sex. A majority of those who reported using a contraceptive method at sexual debut used a male condom (78%) compared to other types of modern contraceptive methods. At the time of survey, 35% of our sexually-experienced unmarried youth sample reported using a modern contraceptive method. There is a statistical difference in modern contraceptive use by age group; in this sample, 38% of the 20–24 year olds reported using a modern method at the time of interview compared to 25% of the 15–19 year olds. Among those who reported modern contraceptive use at time of survey, 63% reported male condom use while 6% reported use of a long-acting reversible contraceptive method (i.e. implant or intrauterine device). There was no age group difference in the type of contraceptive method used.

Table 4 shows the results of the two multivariate cox proportional hazard regression analyses of ‘time to first sex’ and ‘time to first pregnancy’ for our full sample. Adjusted hazard ratios and 95% confidence intervals are shown for the sociodemographic factors, household factors, and family planning knowledge factors and their association with time to first sex (first column of results). We found that those who had secondary education (vs. primary or less education) together with those who reported being strongly religious (vs. somewhat or not religious) were 20–40% less likely to have transitioned to first sex at any given age within our time frame (p<0.05). Unmarried young women who are of the Catholic denomination (vs. other Christians) and are unemployed (vs. employed/student) are 40% more likely to have transitioned to first sex at each age (p<0.05). Those who live in Kisumu are 30% more likely to have transitioned to first sex at each age compared to those in Nairobi (p<0.05). Additionally, young women who know a contraceptive method were three times more likely to have transitioned to first sex at each age than those who do not know of any method (p<0.05). Though those who have a high level of family planning misconceptions are more likely to have transitioned to first sex, this association was not statistically significant (aHR: 1.2; 95% C.I.: 0.9–1.7).

**Table 4:** Cox regression (hazard ratios) of timing of first sex and first pregnancy among never-married females aged 15–24 years in urban Kenya (weighted n = 2020).

	Time to First Sex	Time to First Pregnancy
	aHR (95% C.I.)	aHR (95% C.I.)
**Sociodemographic factors**		
**Education**		
Primary or less education	1.0	1.0
Secondary education	0.6 (0.5–0.8)***	0.4 (0.2–0.6)***
Higher education	0.8 (0.5–1.1)	0.3 (0.1–0.7)**
**Religion**		
Other Christians	1.0	1.0
Catholic	1.4 (1.1–1.7)**	1.0 (0.6–1.6)
Muslim/Others	0.8 (0.5–1.2)	1.2 (0.6–2.3)
**Religiosity**		
Somewhat/not religious	1.0	1.0
Strongly religious	0.7 (0.6–0.9)**	0.8 (0.5–1.1)
**Employment Status**		
Employed/Student	1.0	1.0
Not employed	1.4 (1.1–1.9)**	1.3 (0.8–2.1)
**Household factors**		
**City where household is located**		
Nairobi	1.0	1.0
Mombasa	0.8 (0.6–1.1)	0.5 (0.3–0.9)*
Kisumu	1.3 (1.1–1.7)*	1.5 (1.1–2.2)*
Machakos	0.9 (0.7–1.1)	0.9 (0.5–1.4)
Kakamega	1.2 (0.9–1.5)	1.6 (1.1–2.1)*
**Number of people living in household**		
1–5 persons	1.0	1.0
6 or more persons	0.9 (0.7–1.2)	2.0 (1.3–3.1)**
**Household wealth quintile**		
Poorest	1.0	1.0
Poor	0.7 (0.5–1.1)	1.1 (0.7–1.8)
Middle	0.7 (0.5–1.0)	0.9 (0.5–1.7)
Rich	0.7 (0.5–1.1)	0.6 (0.3–1.2)
Richest	0.8 (0.6–1.0)	0.7 (0.4–1.1)
**Head of household**		
Father Figure	1.0	1.0
Mother Figure	1.0 (0.8–1.3)	1.5 (1.1–2.3)*
Self	1.1 (0.6–1.7)	1.9 (0.8–4.3)
**Duration of residence in household**		
One year or less	1.0	1.0
2–5 years	1.0 (0.7–1.3)	0.8 (0.5–1.3)
Six or more years	0.7 (0.5–1.0)	0.8 (0.4–1.4)
**Family planning knowledge factors**		
**Knowledge of contraceptive methods**		
No	1.0	1.0
Yes	2.9 (1.7–5.0)***	3.4 (1.3–8.8)*
**Family Planning misconceptions**		
Low level (score: 0–2)	1.0	1.0
Medium level (score: 3–5)	1.0 (0.7–1.4)	1.1 (0.7–1.9)
High level (score: 6–8)	1.1 (0.8–1.5)	1.7 (1.1–2.8)*
**Sexual & contraceptive behaviors**		
Early sexual debut (ref = later sexual debut)	—-	2.9 (1.6–4.9)**
Modern contraceptive use at sexual debut (ref = no)	—-	0.7 (0.4–1.2)

aHR: adjusted Hazard Ratio; C.I.: Confidence Interval;

*Statistically significant at p<0.05;

**p<0.01;

***p<0.001

Also included in Table 4 are the adjusted hazard ratios of time to first pregnancy. The risk factors for earlier transition to first pregnancy were residing in Kisumu and Kakamega (vs. Nairobi; aHR: 1.5 and 1.6 respectively); living in a large household (vs. smaller household; aHR: 2.0); living in a mother-headed household (vs. father-headed; aHR: 1.5); knowing at least one contraceptive method (vs. not; aHR: 3.4); having high levels of family planning misconceptions (vs. low level; aHR: 1.7); and having had an early sexual debut (vs. late; aHR: 2.9). However, those who had secondary or high education were 60–70% less likely to have transitioned to first pregnancy at each age compared to primary or less education. In addition, those who live in Mombasa were 50% less likely to transition to first pregnancy at each age compared to those who live in Nairobi. These estimates were statistically significant at p<0.05.

The results of the logistic regression analysis of teenage pregnancy among those aged 20–24 years are presented in Table 5. As shown, those who had secondary or higher education, are of richest wealth category, and used modern contraception at first sex were less likely to have had a teenage pregnancy compared to those with primary or less education, of the poorest wealth category, and did not use contraception at first sex respectively (aOR:≤0.3; p<0.05). The risk factors for teenage pregnancy among 20–24 year olds are living in Kisumu compared to Nairobi (aOR: 3.7) and having had an early sexual debut compared to an older age at sexual debut (aOR: 5.0). These estimates were also statistically significant at p<0.05.

**Table 5:** Logistic regression (odds ratios) of teenage pregnancy among never-married females aged 20–24 years in urban Kenya (weighted n = 1205).

	aOR (95% C.I.)
**Sociodemographic factors**	
**Education**	
Primary education or less	1.0
Secondary education	0.2 (0.1–0.5)***
Higher education	0.1 (0.1–0.4)**
**Religion**	
Other Christians	1.0
Catholic	2.0 (0.9–4.4)
Muslim/Others	2.0 (0.5–8.4)
**Religiosity**	
Somewhat/not religious	1.0
Strongly religious	0.6 (0.3–1.3)
**Employment Status**	
Employed/Student	1.0
Not employed	1.2 (0.5–2.8)
**Household factors**	
**City where household is located**	
Nairobi	1.0
Mombasa	0.5 (0.2–1.4)
Kisumu	3.7 (1.7–8.1)**
Machakos	0.9 (0.4–2.0)
Kakamega	2.3 (0.9–6.0)
**Number of people living in household**	
1–5 persons	1.0
6 or more persons	1.7 (0.6–4.8)
**Household wealth quintile**	
Poorest	1.0
Poor	1.1 (0.4–3.2)
Middle	0.7 (0.2–2.3)
Rich	0.3 (0.1–1.1)
Richest	0.3 (0.1–0.9)*
**Head of household**	
Father Figure	1.0
Mother Figure	1.3 (0.5–3.0)
Self	1.5 (0.5–4.6)
**Duration of residence in household**	
One year or less	1.0
2–5 years	1.0 (0.5–2.0)
Six or more years	0.9 (0.3–3.0)
**Family planning knowledge factors**	
**Knowledge of contraceptive methods**	
No	1.0
Yes	3.9 (0.9–17.5)
**Family Planning misconceptions**	
Low level (score: 0–2)	1.0
Medium level (score: 3–5)	1.9 (0.7–5.2)
High level (score: 6–8)	2.1 (0.8–5.6)
**Sexual & contraceptive behaviors**	
Early sexual debut i.e. ≤14 years at first sex (ref = late sexual debut)	5.0 (1.3–18.5)*
Modern contraceptive use at sexual debut (ref = no)	0.2 (0.1–0.6)**

aOR: adjusted Odds Ratio; C.I.: Confidence Interval;

* Statistically significant at p<0.05;

**p<0.01;

***p<0.001

## Discussion and Conclusion

The findings presented here are important for considering strategies to help young, unmarried women avoid unintended pregnancy and unsafe abortions and, subsequently, reduce maternal morbidity and mortality in urban Kenya. We found that a substantial proportion of our unmarried youth sample are sexually experienced and had ever been pregnant. The short time lapse between first sex and first pregnancy reflects the contraceptive needs of these unmarried young women. Although a small proportion of these women reported modern contraceptive use at the time of survey, a majority of the users reported male condom use as their current method. Given that this is a coitus-based method together with the potential effort required to convince the male partner to use, especially in a power-imbalanced couple dynamics, the contraceptive coverage of male condoms for these young women may be lower than measured. Only a small proportion of unmarried young women reported use of long-acting reversible contraceptive methods (such as implants and intrauterine devices); there methods are known to provide more effective contraceptive coverage and require little effort on the user.

Our multivariate findings show that among unmarried young females, those with at least secondary education are less likely to transition to first sex and pregnancy at each age. Moreover, while Catholic youth are more likely than other Christian youth to transition to first sex at each age, those youth who are strongly religious, no matter the religion, are less likely to transition to first sex at each age. This suggests that programs should consider incorporating religiosity since it is protective for early sexual debut. We also demonstrate a number of household factors associated with increased risk of pregnancy. In particular, women from larger households and mother-headed households were more likely to have ever been pregnant at each age than their counterparts not living in these types of households. Finally, poor reproductive health information (e.g., family planning myths and misconceptions) and early sexual debut (first sex at age 14 or below) were associated with greater risk of pregnancy at each age. The positive association between knowledge of contraceptive methods and transition to first sex and first pregnancy likely reflects the post-experience effect. That is, women become contraceptive knowledgeable after they have initiated these sexual and reproductive behaviors. There is a need for sexuality education for younger adolescents so that prior to their first experiences, they are equipped with that appropriate information on safer sexual practices. Our analyses among the older youth (ages 20–24) also examined factors associated with the experience of a teenage pregnancy. Women who had early sexual debut and women who did not use contraception at first sex were significantly more likely to have had a teen pregnancy than their counterparts with later first sex and protected first sex. In addition, higher education was found to be negatively associated with having a teen pregnancy.

Our study applies the Problem Behavior Theory developed by Jessor to examine factors associated with sexual initiation, pregnancy experience, and teenage pregnancy experience among unmarried young females in urban Kenya. [26, 27] As shown by Jessor, factors at multiple levels act as protective and enabling influences on youth risk-taking. In particular, individual factors such as education and religiosity are protective factors, whereas, household factors such as being poor, larger household size, and living in a female-headed household are enabling factors for transition to pregnancy and teen pregnancy experience. Young, unmarried women living in large households, female-headed households, or poorer households may have less supervision over their actions since it is likely that household members are working or occupied with caring for others in the household. Moreover, each of these household factors is related to poverty, which might lead young women to offer sexual services for cash or other goods, increasing their risk of a pregnancy. [12, 13]

Previous studies on early sexual debut and early or teen pregnancy experience from Africa that use nationally representative data found similar results to those presented here. [10, 23, 28, 29, 37, 38] Moreover, recent studies from the slums of Nairobi also show high rates of early sexual initiation [9, 22, 25], early pregnancy [22, 25], and unintended pregnancy [7, 25] among young females. Our study contributes to the existing literature by examining urban youth from multiple settings in Kenya and by using a large sample of unmarried young females to examine premarital sexual activity, premarital contraceptive use, and premarital pregnancy experience. We demonstrated that young women in Kisumu are more likely to transition to first sex and first pregnancy and have almost four times the odds of having a teen pregnancy than those in Nairobi. Therefore, programs in Kisumu need to target young women to consider how to ensure that sexual activity is protected using condoms and other family planning methods to avoid STI and unintended pregnancies.

It is worth noting that the majority of pregnancies among unmarried youth are reported as unintended. This demonstrates important gaps in family planning programs to serve unmarried, sexually active youth. It is possible that there are barriers to use of modern contraceptive methods at the facility level e.g. providers refuse to offer methods to youth or require parental consent. [39] Programs are needed to prevent these early and teen pregnancies to ensure that all pregnancies are intended and reduce the recourse to abortion.

This study is not without limitations. First, because about a third of the sample is between the ages of 15–19, these youth have not yet had a chance to transition to first sex or first pregnancy. Thus, estimates of mean (or median) age of first sex, first pregnancy and duration between first sex and pregnancy are biased by those youth who have already transitioned to these behaviors. However, since the MLE project is a longitudinal study with a midterm and endline follow-up at two year increments, many of these young people will make these transitions by endline and it will be possible to examine these means and medians at a later date. Second, these data are cross-sectional and thus it is difficult to show causality. For example, it is possible that women developed the myths and misconceptions after their first sex or first pregnancy but with the available data it is not possible to determine the appropriate direction of the effect. In addition, while we try to use variables in the model that would come “before” the outcomes, it is possible that some are still in transition and thus may have been influenced by the outcome. For example, for the level of education, those women who became pregnant may have terminated their education early and thus the pregnancy influences the low education rather than low education influencing the earlier age of pregnancy. Finally, this study uses self-reported information on sexual behaviors (age at first sex, age at first pregnancy, and contraceptive use at first sex); young women might under (or over) report these behaviors. [40, 41] Interviewers were trained to be sensitive to reporting among women of all ages which may have helped to reduce some of this bias.

To conclude, unmarried young women in urban areas are at risk of STIs and unintended pregnancy, especially in the window between sexual initiation and delayed marriage. Identifying strategies to ensure that young women have knowledge of family planning methods, access to these methods, and can afford to use them is important to improve young women’s health and well-being. Programs for unmarried youth can be undertaken in various settings including schools, markets, and other settings where young people congregate. Outreach workers or peer educators can provide information, counseling, and methods (spacing methods) on site and as needed refer young women for long-acting and permanent methods. Program strategies may need to differ by setting. For example, in Kisumu where teen pregnancy is common, programs are needed to offer family planning methods to all young, unmarried women who are sexually active or intending to become sexually active. Moreover, in Kisumu, dual protection should be promoted since it is the site in Kenya with some of the highest HIV prevalence rates. [42] Programs that target enabling and protective factors of youth sexual and reproductive health are crucial for improving young people’s long-term potential by avoiding early and unintended pregnancies and reducing maternal morbidity and mortality.

## Supporting Information

S1 Table Family planning misconception. (DOCX)
