# Supplementary material for: Determinants of Sexual Activity and Pregnancy among Unmarried Young Women in Urban Kenya: A Cross-Sectional Study
Source: PLoS One. 2015 Jun 5;10(6):e0129286. doi: 10.1371/journal.pone.0129286 (PMC4457813; doi:10.1371/journal.pone.0129286)
Supplement: S1 Table — (DOCX) [file pone.0129286.s001.docx]

**Supporting Information**

**S1 Table. Family planning misconception**

| **Survey Question** | **Family planning misconception statements** |
| --- | --- |
| 379 | Use of a contraceptive injection can make a woman permanently infertile |
| 380 | People who use contraception end up with health problems |
| 381 | Contraceptives can harm your womb |
| 382 | Contraceptives reduce women’s sexual urge |
| 383 | Contraceptives can cause cancer |
| 384 | Contraceptives can give you deformed babies |
| 385 | Contraceptives are dangerous to your health |
| 386 | Women who use family planning/contraception may become promiscuous |
| Responses are on a 4-point Likert scale – strongly disagree, disagree, agree, and strongly agree.  Responses were recoded to ‘1’ if strongly agree or agree and ‘0’ if strongly disagree or disagree.  Scoring scale for family planning misconceptions: 0 – 8. | |
